# Distinct Evolutionary Selection Patterns in Temperate and Filamentous Phages of Phocaeicola vulgatus in Health and IBD

**DOI:** 10.21203/rs.3.rs-10845320/v1

**Published:** 2026-09-16

**Authors:** Megan Baldridge, Danielle Campbell, Xiaofen Wu, Olivia Emerson, Haina Jin, Sarah Grambo

**Affiliations:** Washington University in St. Louis; Washington University in St. Louis; Washington University in St. Louis; Washington University in St. Louis; Washington University in St. Louis; Washington University in St. Louis

## Abstract

The gut virome is profoundly altered in inflammatory bowel disease (IBD), though phage evolution during inflammation remains poorly understood. We used viral tagging (VT) to recover phages of *Phocaeicola vulgatus*, a gut-associated pathobiont, from healthy and Crohn’s disease (CD) viromes, revealing phages related to the tailed phage BV01, and the first filamentous phage (PvIno1) of any *Bacteroidaceae* host. BV01-like phages accumulated point mutations in tail genes during health, diversity which was lost in CD, while the configuration of a tail-fiber shufflon varied with disease state. In PvIno1, diversification was concentrated to a regulatory region, rather than structural genes as in BV01-like phages. Together, these findings indicate that inflammation imposes unique evolutionary pressures on distinct phages types. Integrating evolutionary dynamics with current ecological models of the IBD phageome is a critical step toward designing phage-based therapeutics for gut dysbiosis and understanding the complex interactions between phages, bacteria, and human hosts.

## Introduction

The human gut phageome is significantly altered in inflammatory bowel disease (IBD)—manifesting primarily as Crohn’s disease (CD) and ulcerative colitis (UC)—where temperate phages dominate over the lytic phages characteristic of the healthy gut^1–5^. These shifts reflect the ecological strategies required to survive host inflammation, and while this macro-scale perspective of the IBD phageome is well documented, the effects of inflammation on phage evolution remain largely unexplored.

The vast majority of gut phages lack known bacterial hosts, presenting a critical barrier to functional understanding of the microbiome. Host identification has largely relied on computational predictions from metagenomic data or *in vitro* culture, which is limited to phages and hosts amenable to lab conditions^6–9^. High-throughput methods, such as viral tagging (VT), bridge this gap by isolating physically interacting phage-bacteria complexes^10–14^. Our approach applies VT to a single host with a predefined genome and sorts thousands of cells in bulk, streamlining analysis and deeply surveying host-defined phage populations.

Here we applied VT to *Phocaeicola vulgatus* (formerly *Bacteroides vulgatus*), a ubiquitous gut microbe that functions as a critical pathobiont in IBD via multiple mechanisms^15–28^. Temperate phages of *Phocaeicola* and other *Bacteroidaceae* profoundly influence host biology. In *P. vulgatus*, the transposable phage Hankyphage encodes host capsular polysaccharide (CPS) synthesis loci^29,30^, while prophage integration of phage BV01 disrupts a key bile acid sensor, broadly altering the transcriptome and downstream metabolism^31^. These complex interactions make *P. vulgatus* an ideal model system to investigate viral dynamics in IBD. We hypothesized that, beyond driving macro-scale ecological shifts, inflammation actively shapes the ongoing evolution of these phages.

We employed VT to survey *P. vulgatus* phage populations in health and CD, defining 43 vOTUs that could not be assembled by virome sequencing alone, highlighting the strength of VT for capturing low abundance species. We focused on two VT-detected vOTUs: one temperate tailed phage vOTU closely related to BV01 and one filamentous phage vOTU, the first to be described for a *Bacteroidaceae* host. Our bulk VT approach enabled analysis of within-population mutation at nucleotide-level resolution, quantification of phage lifecycle activities, and the description of an active phage-encoded shufflon affecting a BV01-like phage tail fiber gene. Our results indicate that CD exerts unique selective pressures on both phage types. By integrating viral evolution into existing ecological paradigms of the inflamed gut, this work establishes a necessary foundation for designing future phage-based strategies to remediate gut dysbiosis.

## Results

### Viromes, VT & enrichment methods sample unique phage populations

**Figure 1:**
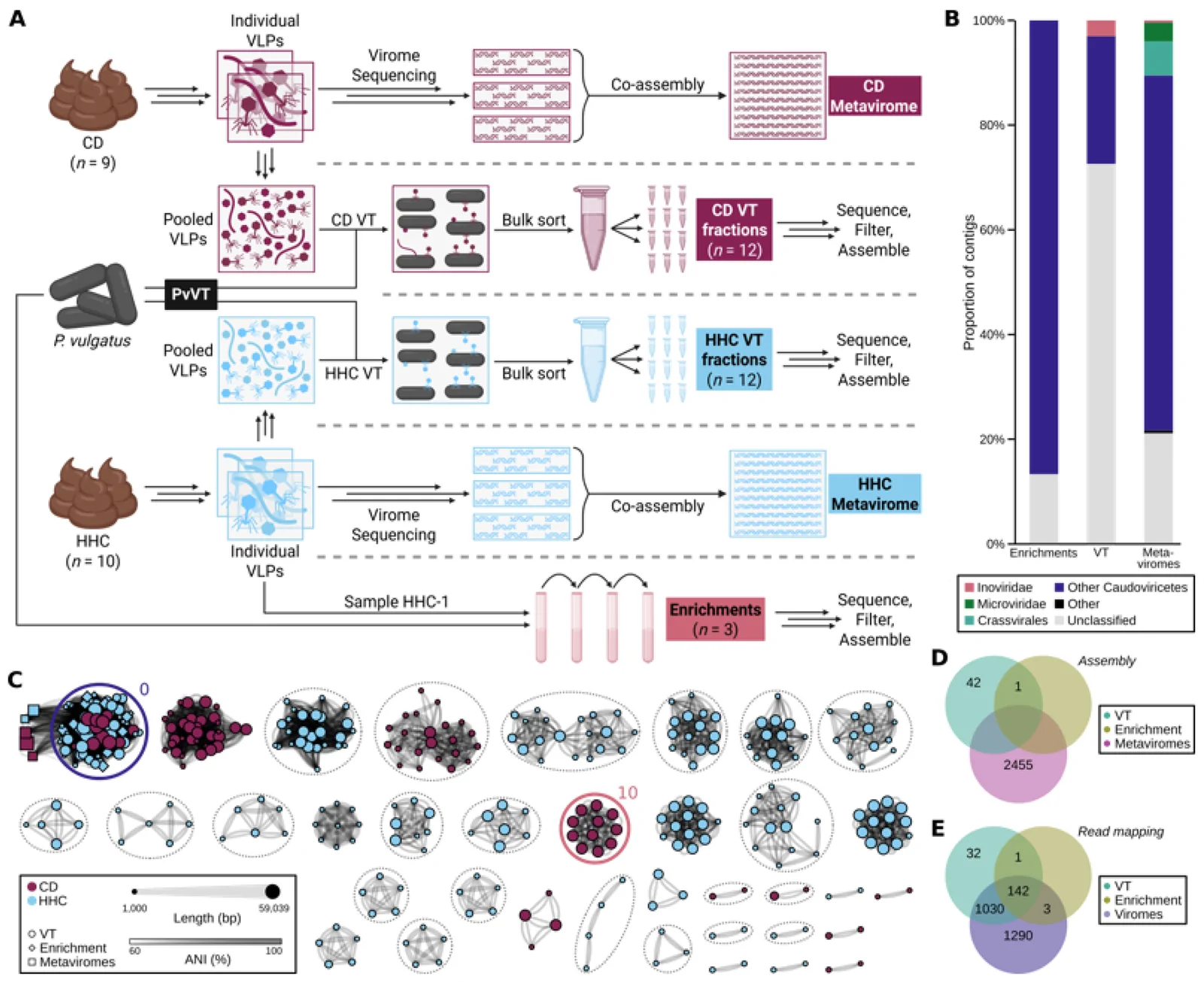
Workflow and multi-method diversity analysis. **(A)** Schematic of experimental workflow for metaviromes, VT and enrichments. **(B)** Taxonomic classification of viral contigs by detection method using geNomad^34^. Full taxonomy is shown in Fig S1D. **(C)** Nucleotide similarity network based on average nucleotide identity (ANI) calculated through vClust^73^. Only contigs assembled from VT, enrichments, or directly adjacent metavirome contigs are shown. Dotted circles indicate vOTUs with at least one member contig with a viral score >50% by geNomad^34^. Nine vOTUs comprised of a single contig are not show. The network was visualized in Cytoscape^74^. **(D)** Count of vOTUs shared between detection methods in assemblies. **(E)** Count of vOTUs shared between detection methods by read mapping. vOTU presence required a TPM ≥1.0 and coverage breadth ≥50% of the largest vOTU contig.

To comprehensively profile the *P. vulgatus* phageome, we performed viral tagging (VT) using virus-like particle (VLP) pools from Crohn’s disease (CD) and matched healthy household control (HHC) samples alongside *in vitro* enrichment of phages from one cohort sample (Fig. 1A, **Table S1**). We also employed previously assembled VLP pool metaviromes, the virome for sample HHC-1^10^, and a re-sequenced HHC-1 virome at the time of enrichment. Quality filtering to remove host, human, and adapter sequences revealed significantly lower read retention in CD compared to HHC VT samples (2.5% ± 0.2% and 22.4% ± 4.6%; Student’s t-test, *p* = 1.5 × 10^−7^; **Fig. S1A**), driven by higher proportions of *P. vulgatus* host sequence. However, this ~ 18-fold difference in retained reads yielded only a ~ 2-fold difference in total assembly length (**Table S2**), which was driven by lower sequence diversity of CD VT samples (**Fig. S1B**). By comparison, source metaviromes yielded larger assemblies (4.7 Mb for CD; 7.0 Mb for HHC) with greater nucleotide diversity than VT samples (**Fig. S1C**), establishing a baseline for cross-method comparisons. Finally, *in vitro* enrichments and resequencing of the HHC-1 virome was performed at low depth, yielding small assemblies (**Fig. S1A**, **Table S2**).

Clustering with vConTACT2^32^ and a relaxed BLASTn^33^ approach against Viral RefSeq revealed that 14 and 516 contigs, respectively, all originating from metaviromes, matched known phages, including *Microviridae*, crAssphages, and *Caudovirales* phages such as Bacteroides phages B40-8 and B124-4 (relatives of Phocaeicola phage BV01)^31^. Notably, despite *P. vulgatus* being a known host for phages across these lineages, zero VT or enrichment contigs matched reference databases, demonstrating that VT captures uncharacterized viral space. Marker- and sequence-based classification using geNomad^34^ assigned viral origin to 73.3% of enrichment (*n* = 11), 12.3% of VT (*n* = 45), and 56.7% of metavirome contigs (*n* = 1,699) (Figs. 1B, **S1D; Table S3**). While metaviromes were dominated by *Caudoviricetes*, *Crassvirales*, and *Microviridae*, VT strongly enriched for filamentous phages (*Inoviridae*; *n* = 11, 3.0% of VT contigs vs. 0.4% in metaviromes)—representing the first described filamentous phages for *P. vulgatus* or any *Bacteroidaceae* host.

GeNomad^34^ also identified non-viral elements, predicting plasmids in enrichment (*n* = 4) and VT (*n* = 36) assemblies, alongside minor *Staphylococcus aureus* contamination in enrichments (*n* = 2). The remaining unclassified VT contigs (*n* = 282) ranged from encoding isolated phage- or plasmid-like genes to lacking functional annotations entirely, likely reflecting a mix of uncharacterized mobile elements, multiple displacement amplification (MDA) artifacts, and chimeric assemblies. To evaluate overall viral diversity independent of gene annotations, all contigs were clustered at the vOTU level (95% ANI). Across the dataset, VT and enrichment contigs condensed into 43 vOTUs (Fig. 1C, **Table S3**). Method and source condition exclusivity at the assembly level was nearly absolute, with only a single vOTU (vOTU_0) shared between methods (VT and enrichment) and disease conditions (CD and HHC VT) (Figs. 1C, 1D). Notably, 25 of these 43 clusters contained member contigs with a geNomad viral confidence score above 50%.

Within VT samples, read mapping revealed lower phage diversity in CD compared to HHC, with collector’s curves plateauing at ~ 32 vOTUs for HHC versus a lower trajectory (≤ 20 vOTUs) for CD (**Figs. S1E, S1F**). To test whether un-assembled vOTUs were truly absent or simply below assembly coverage thresholds, we cross-mapped reads between VT conditions (**Fig. S2A**). This revealed that 17 vOTUs (39.5%) were actually present in both CD and HHC samples, reducing CD-exclusive vOTUs from 13 to nine and HHC-exclusive vOTUs from 29 to 17. Expanding this cross-mapping approach across experimental methods and input viromes similarly uncovered extensive shared membership (Figs. 1D, 1E): enrichment reads mapped directly to four VT-derived vOTUs, 10 VT- or enrichment-exclusive vOTUs were detected in source virome reads, and 1,165 metavirome-assembled vOTUs were detected within VT and enrichment datasets. The low detection levels for some cross-mapped vOTUs likely indicate background noise or sharing of motifs between vOTUs. Together, these results demonstrate that low abundance rather than biological absence drives apparent exclusivity across cohorts and methods, highlighting VT’s capacity to capture low-abundance phages missed by other methods.

To evaluate the broader ecological prevalence and clinical relevance of these vOTUs, we queried an independent benchmark dataset of 169 published CD, ulcerative colitis (UC), and HHC viromes^3^. Consistent with our primary cohort, VT-derived vOTUs were detected at low relative abundance across external samples (**Fig. S2A**). Although no statistically significant differences in vOTU abundance were observed within our primary dataset—likely owing to sample size constraints—the increased statistical power of the external cohort identified two VT-derived vOTUs that differed significantly across CD, UC, and HHC conditions (**Fig. S2B**). While both differential vOTUs currently lack functional annotations, these results demonstrate that VT captures novel disease-associated phage diversity that remain undetectable in virome assemblies.

Ultimately, these findings demonstrate how methodological selection shapes viral recovery. While standard viromics provides a broad profile of dominant phages, VT surveys ultra-low-abundance viruses by targeting a specific host, and enrichment imposes an even stricter selection requiring active replication. Because many VT-captured contigs lacked deep functional annotations, we focused our subsequent characterization on the two highest-confidence vOTUs identified across both targeted approaches: vOTU_0, a BV01-like temperate tailed phage, and vOTU_10, a novel filamentous phage (Inoviridae), hereafter designated PvIno1.

### BV01-like phage microevolution is distinct between Crohn’s disease and healthy populations

**Figure 2:**
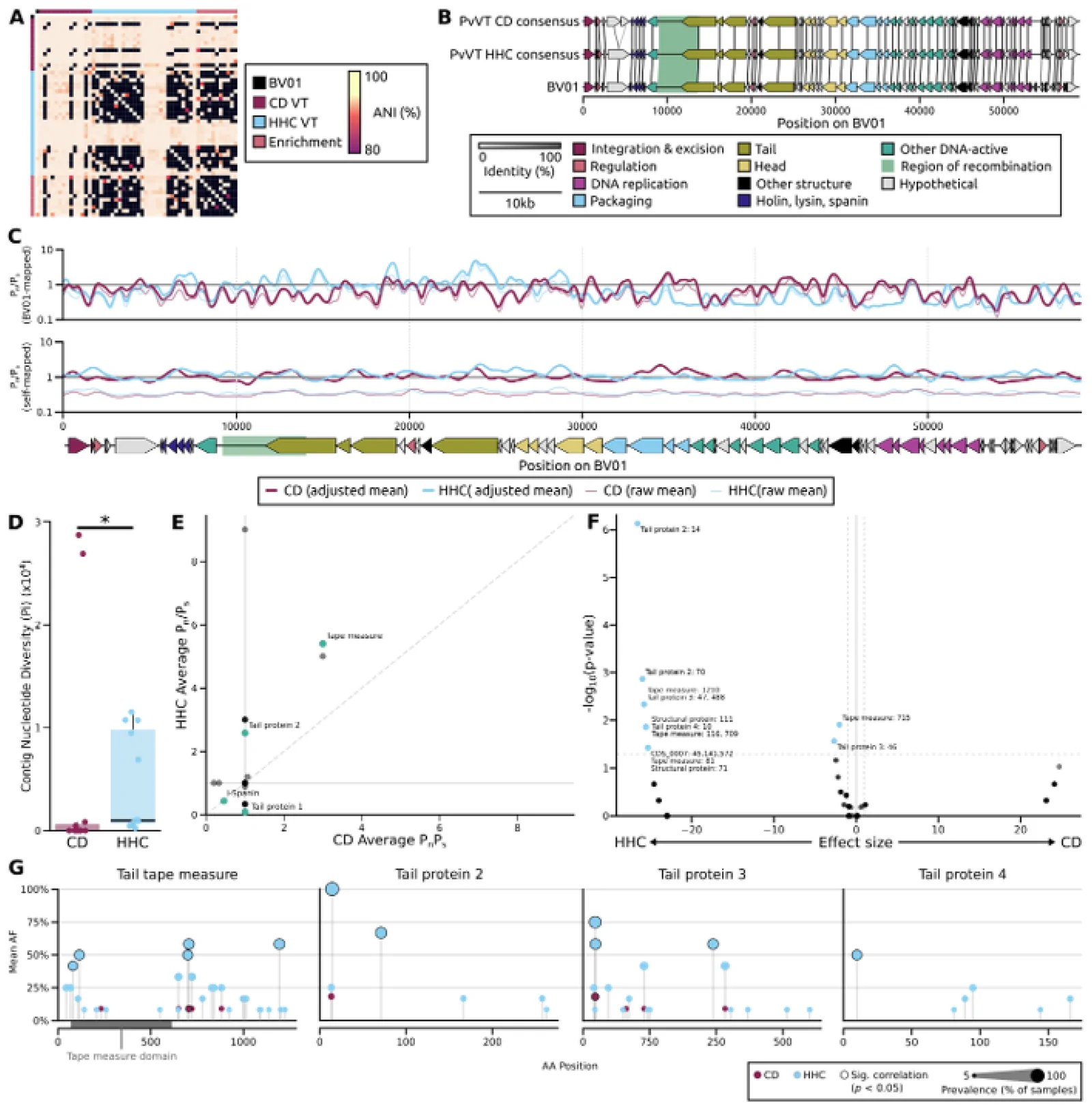
BV01-like phage microdiversity varies between CD and HHC. **(A)** Average nucleotide identity (ANI) between the BV01 reference genome and all assembled vOTU_0 contigs. ANI was calculated as the length-weighted ANI of reciprocal local alignments >2 kb based on reciprocal BLASTn^33^ outputs. **(B)** Protein similarity alignment of the BV01 reference sequence and the CD and HHC VT consensus contigs with annotations. Consensus contigs were assembled from pools of all CD and HHC VT reads using the same assembly methods as for individual VT samples. Alignments were generated and visualized with Clinker^71^. **(C)** Site-by-site ratio of nonsynonymous to synonymous polymorphisms (*P*_*n*_*/P*_*s*_) across the length of the BV01 genome (59,039 bp) was calculated for direct BV01 reference-mapped and for self-mapping to individual vOTU_0 contigs (*n* = 73). Self-mapping to individual contigs was mapped *post hoc* to BV01 genome coordinates. Adjusted means were calculated as a sliding-window mean across 400 bp windows with a 40 bp step size restricted to active variant sites (N_mutations_ > 0 or S_mutations_ > 0). Curves were smoothed by a 1,000 bp window Gaussian transformation. **(D)** Nucleotide diversity (*π*) of CD (*n* = 11) and HHC (*n* = 12) sample reads compared to the BV01 reference genome. Asterisks denote statistically significant pairwise differences calculated via uncorrected two-sided Mann-Whitney U test (* *p* < 0.05). Data are presented as median (horizontal), 25th-75th interquartile range (box), ± 1.5 × IQR (whiskers), and individual sample values (points). **(E)** Average *P*_*n*_*/P*_*s*_ was calculated for each gene (*n* = 84) in CD and HHC from BV01-mapping data. Highlighted and labeled genes exhibit significant divergence from neutral selection (*P*_*n*_*/P*_*s*_ = 1) in HHC VT samples. A pseudocount of 0.05 was applied to all genes. No genes were significantly different from neutral selection in CD VT. Significant genes were identified by Wilcoxon signed-rank test with Benjamini-Hochberg False Discovery Rate multiple testing corrections *q* < 0.05. **(F)** Volcano plot of effect size and significance of all nonsynonymous mutations (*n* = 228). Each point represents a unique SNP, and is labeled its amino acid locus. Statistical significance was calculated using a two-sided Fisher’s exact test. Effect sizes are expressed on the horizontal axis as the log_2_-fold change between CD and HHC. Highlighted points indicate mutation sites crossing a nominal significance threshold of raw *p* < 0.05. **(G)** Lollipop plots depicting mean allele frequency across samples and sample prevalence for tail proteins with sites determined to be significant (Fig 1F). Blue indicates alleles in HHC; maroon indicates alleles in CD. Only nonsynonymous SNPs are shown.

The largest vOTU, vOTU_0, comprised 73 contigs, all annotated as temperate tailed phages (**Table S3**). It was detected across all VT and enrichment assemblies at exceptionally high abundance (**Fig. S2A**). vOTU_0 phages were closely related (ANI > 99.7%) to the temperate phage BV01 (family *Salyersviridae*) (Fig. 2A), which was missed during initial automated classification due to its absence in reference databases. Phylogenetic analysis using six conserved proteins further confirmed this close relationship (**Fig. S3A**), while protein similarity alignments of BV01 and vOTU_0 contigs demonstrated complete synteny and functional gene conservation (Figs. 2B, **S3B**).

Five metavirome contigs were found to be similar to, but not clustered with, vOTU_0, representing the only contig-level similarity between metaviromes and VT or enrichments (Fig. 1C). Despite their presence in input viromes, these contigs did not interact with *P. vulgatus* during VT, potentially due to their distant relationship with BV01 and other salyersviruses (**Figs. S3C, S3D**). To distinguish whether the failure to recover these vOTU_0-similar contigs by VT reflected true biological host specificity or random non-detection, we mapped reads from individual cohort viromes and VT samples (Table 1). In input cohort viromes, the five vOTU_0-similar contigs were 86-fold more abundant than vOTU_0 contigs. In VT samples, this ratio completely inverted: vOTU_0 contigs became 887-fold more abundant than the vOTU_0-similar contigs (*p* < 0.0001, Poisson distribution). This dramatic shift confirms that the exclusion of vOTU_0-similar contigs during VT was driven by host strain specificity rather than low starting abundance in input VLP samples, validating VT’s high selectivity and minimal background binding.

To evaluate intra-population microdiversity beyond consensus contigs, we performed single nucleotide polymorphism (SNP) analysis across VT samples. This approach treats each sample as a mixed population of phages, which is consistent with each containing many sorted cells, and highlights VT’s utility for studying biologically relevant population dynamics. Because vOTU_0 dominated VT samples from both conditions (79.5% ± 2.9% relative abundance in CD VT; 45.9% ± 7.5% in HHC VT), read depth across the BV01 reference genome was robust (102.6× ± 128.1× in CD VT; 231.6× ± 237.6× in HHC VT; 100% of samples ≥ 20× depth) with near-complete genome coverage (100% in CD VT; 99.6% ± 1.0% in HHC VT) (**Figs. S1B, S1E, S1F**). In contrast, read coverage across both input cohort viromes and the external dataset of 169 viromes^3^ was insufficient to support variant calling (**Figs. S4A, S4B**). Despite high contig sequence identity, SNP profiling revealed that vOTU_0 populations are non-homogeneous and exhibit variable evolutionary pressures across the genome (Fig. 2C). *P_n_/P_s_* ratios calculated by self-mapping reads to sample-matched vOTU_0 contigs were lower than those calculated against the BV01 reference, reflecting the distinction between short-term intra-population microevolution and long-term divergence. Overall, vOTU_0 populations from HHC VT samples displayed significantly higher nucleotide diversity than those from CD VT (Fig. 2D).

Beyond the contig level, no individual genes differed significantly in their *P_n_/P_s_* values in direct comparisons between CD and HHC VT conditions. However, when examining gene-level selection against neutrality (*P_n_/P_s_* = 1), several proteins were identified for HHC. The inner membrane spanin exhibited significant purifying selection, reflecting its conserved requirement for mechanical interactions during host lysis (Figs. 2E, **S4C**). Three additional genes—tail protein 1 (purifying selection), tail protein 2, and the tail tape measure protein (both under diversifying selection)—deviated significantly from neutrality (Figs. 2E, **S4C**). This diversifying selection in tail components suggests an active phage-host coevolutionary arms race^35^, a pattern further supported by elevated nucleotide diversity and mutation density in these genes, where tail protein 3 was also significant (**Figs. S4D**, **S4E**). At the single-nucleotide level, 17 nonsynonymous SNPs (yielding 16 amino acid changes or frameshifts) were significantly enriched in BV01-like phages from HHC VT samples (Fig. 2F). Except for three SNPs in an unannotated gene, all HHC-associated mutations occurred in structural genes: tail proteins 2, 3, and 4, the tail tape measure, and an unspecified structural protein. Within the tape measure protein, SNPs are largely excluded from the conserved tape measure domain, demonstrating differential evolutionary pressures over the length of the gene based on functionality (Fig. 2G). Most notably, a substitution in tail protein 2 (Pro14Ser) was fixed in HHC populations (Fig. 2G), reflecting strong, condition-specific selection at this position. Remarkably, no significant gene- or SNP-level patterns were associated with CD, indicating that BV01-like phages experience purifying selection in the inflamed intestine, which starkly contrasts the diversifying selection imposed specifically on the tail machinery during health.

**Figure 3:**
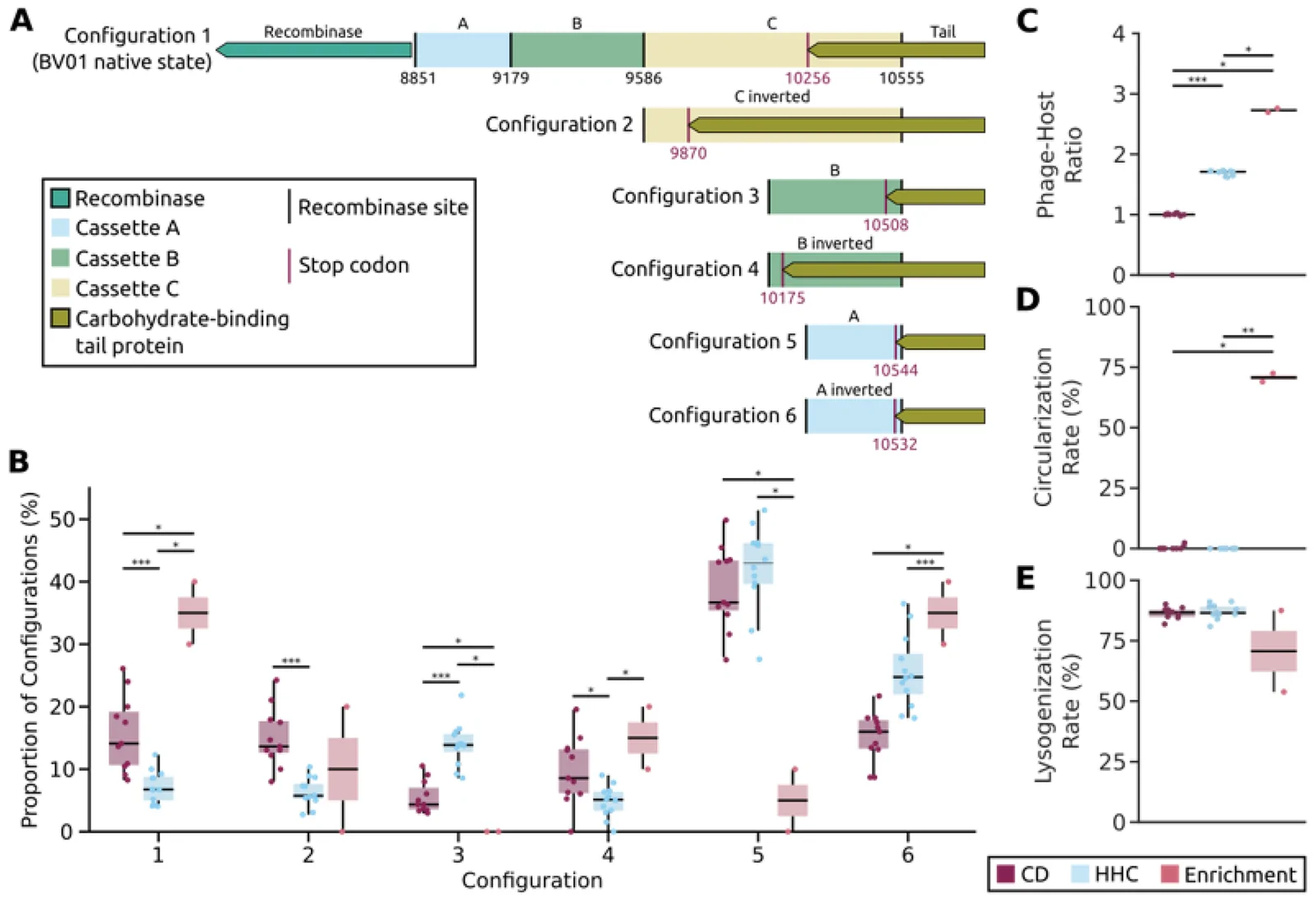
Dynamics of the tail protein 2 shufflon and phage-host interactions for BV01-like phages. **(A)** Schematic overview of the possible structural configurations of the shufflon and each configuration’s effects on the tail protein 2 coding sequence and stop position. **(B)** Relative abundance of individual shufflon configurations in CD VT, HHC VT, and enrichments. Abundance was determine by normalizing read counts for those spanning the individual directional cassette-tail gene junctions to the total reads at the junction. **(C)** Phage-host genomic copy number ratios were calculated from average sequencing depth of phage BV01 (as a proxy for individual vOTU_0 phage contigs) and the *P. vulgatus* ΔBV01 genome. One 2,367-bp region of homology between the phage and bacterial chromosome (equivalent to NC_009614.1: 3,904,942-3,97,308) was masked during read mapping. A copy number ratio of approximately 1 indicates a stable, single-copy integrated prophage population. **(E)** Prophage integration frequency (lysogeny rate) at the 25-bp *attB* site was calculated from discordant read pairs and chimeric reads spanning the *attL* and *attR* sites relative to that of the wildtype *attB* site. **(D)** Phage circularization rate was computed as the ratio of discordant read pairs and chimeric reads spanning the *attP* site to the total coverage depth within 2-kb terminal windows. **(B-E)** Asterisks denote statistically significant pairwise differences calculated via uncorrected two-sided Mann-Whitney U test (* *p* < 0.05, ** *p* < 0.01, *** *p* < 0.001). Data are presented as median (horizontal), 25th–75th interquartile range (box), ± 1.5 × IQR (whiskers), and individual sample values (points).

Beyond point-mutation sweeps, genomic alignments revealed complex structural rearrangements associated with another tail component gene, an annotated carbohydrate-binding tail fiber protein (Figs. 2B, **S3B**). Assembled BV01-like contigs exhibited recombination and inversion densely concentrated within a 1.7 kb window flanked by a recombinase and overlapping with the distal end of the tail fiber gene—an architecture strongly reminiscent of an active multi-cassette shufflon (Fig. 3A). Detailed sequence analysis supported this classification, identifying directional 10-bp motifs (TGGGAGAACT) with conserved 14–18 bp terminal extensions that separate three non-homologous cassettes, likely serving as recognition sites for recombination. Because the shufflon overlaps the tail fiber gene, cassette inversions introduce unique stop codons that yield six distinct protein variants with unique sequences and lengths (Fig. 3A). VT read analysis confirmed shufflon activity in BV01-like phage populations captured during VT, with all possible configurations detected across CD and HHC samples, although at significantly different frequencies (Fig. 3B). Within enrichments, configurations 1 (the native BV01 configuration) and 6 dominate, suggesting that specific tail fiber types may contribute to infection and maintenance *in vitro*. Between CD and HHC VT, the relative abundance of four configurations varied significantly, suggesting the gut environment also influences tail fiber variation. Input viromes lacked reads mapping to the recognition site at the tail fiber-cassette junction. Within the large external cohort^3^, however, this junction was detected in 2.4% of samples at low rates, but confirmed configurations 1, 3, and 4 alongside four additional unique sequences, suggesting other cassettes can occur in broader populations.

BV01 has never been successfully cultivated^31^, and we therefore did not expect to observe active infection in our experiments. However, *in vitro* enrichments exhibited high BV01-like phage-host ratios, circularization rates (indicating replication or extracellular phage genomes), and lysogenization that demonstrated active infection of *P. vulgatus* (Figs. 3C, 3D, 3E). Furthermore, high integration rates occurred in all VT samples, which exclusively occurred at the known BV01 integration site^31^. Unlike enrichments, phage circularization was largely undetectable during VT, indicating a strong preference for integration rather than replication or maintenance of the virion state. Together, these findings demonstrate that vOTU_0 phages possess robust infectivity toward *P. vulgatus*, suggesting that SNPs and shufflon variants may overcome the cultivation barriers previously observed for the reference BV01 phage.

#### *Structural conservation in active filamentous phages of* P. vulgatus

**Figure 4:**
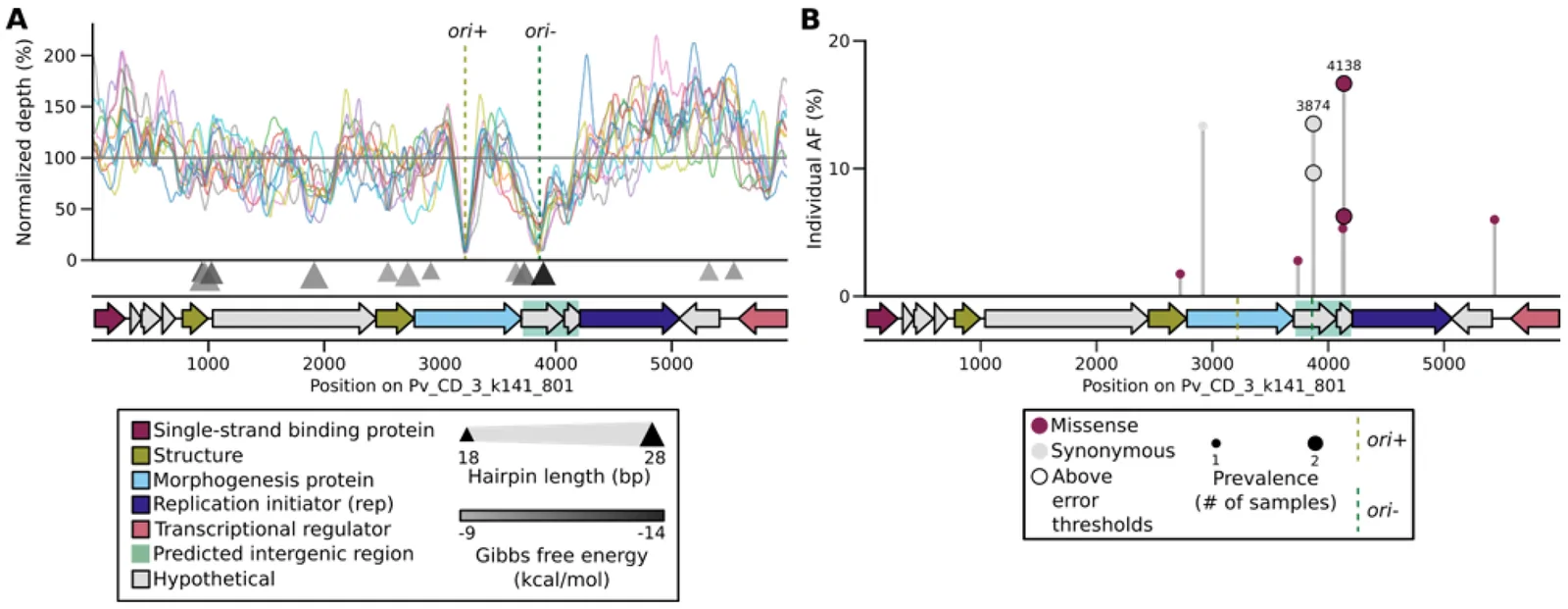
PvIno1 structural features and microdiversity. **(A)** Annotation of the vOTU representative contig (Pv_CD_3_k141_801) and normalized depth profile by individual sample read mapping. Depth at each site was normalized to mean depth over the full contig length for that sample. Each trace represents a different sample (*n* = 11). **(B)** Lollipop plot of SNP allele frequency (AF) within individual samples (*n* = 11) mapped to the vOTU representative contig. Stem heights depict the maximum AF observed at that site within a single sample. SNPs detected within two samples show two points on the same stem. Points labeled as “above error thresholds” occur in two samples with allele frequency (AF) > 1%.

VT identified 11 filamentous phage contigs (family *Inoviridae*; “PvIno1”) comprising vOTU_10 (Fig. 1C) and exclusively originating from CD VT samples (100% sample prevalence) with remarkable sequence conservation (ANI > 99.85%; Figs. 1C, 4A, **S5A**). PvIno1 phages demonstrated a consistent ~ 5,994 bp length, encoded 13–14 genes, including core inovirus structural, replication (Rep), and morphogenesis proteins (Figs. 4A, **S5B**). Read mapping confirmed a > 58,000-fold enrichment in CD relative to trace background mapping in HHC VT samples (*p* < 0.0001, Mann-Whitney U test; Table 2, **Fig. S2A**). Phage-host ratios indicated that 47.3% ± 7.1% of *P. vulgatus* cells harbored PvIno1, a value potentially deflated by MDA biases toward small circular DNA^36–39^. Rather than generating stochastic noise, MDA amplification yielded a strikingly reproducible coverage topology across the PvIno1 genome in all samples (average pairwise *R* = 0.56 ± 0.13) (Figs. 4A, **S5B**).

Normalized depth profiles revealed two major depletion sites which we hypothesized correspond to the positive- and negative-strand origins of replication (*ori +* and *ori−*) (Figs. 4A, **S5B**). The primary depletion site (98.5% ± 2.1% depth depletion) occurred within a 22-bp window centered at a position that aligns with the initial assembly ends (prior to contig rotation). Raw read counts at this locus dropped to 0–2 reads across all 11 CD VT samples (Figs. 4A, **S5C**). This pronounced depletion is suggestive of a putative *ori+* site where rolling circle replication (RCR) is initiated by a single-strand nick, creating a physical barrier during MDA. Despite this nick site, circularization rates averaged 42.9% ± 5.0%, while the remaining 57.1% existed as unlinked sites, a signature of free termini on linear concatemer intermediates generated during RCR. Together, this depletion site and the abundance of linear intermediates point to active vOTU_10 replication within host cells.

The second depletion site identified (81.0% ± 12.6% depth depletion) occurred within a 71-bp window and is suggestive of the *ori−*, as it occurs alongside several strong hairpin loops (Figs. 4A, **S5C**). These structures are required for RNA polymerase binding and primer synthesis, however they also cause Phi29 polymerase stalling and hinder random hexamer priming, resulting in localized reduction in sequencing depth^40–42^. While the *ori−* characteristically occurs in an intergenic region between the morphogenesis and *rep* genes, in PvIno1 it mapped within an unannotated gene that is nonetheless closely flanked by the morphogenesis and *rep* genes. We reason that one or both of the unannotated genes in this region may be false predictions, placing the *ori−* in its canonical intergenic locus.

SNP analysis found low nucleotide diversity for vOTU_10 phages (9.4 × 10^− 5^ ± 3.7 × 10^− 5^) comparable to that of BV01-like phages (Fig. 2D). Reflecting their compact genome size, nine total SNPs were identified at seven sites across five samples; all SNPs were unique to individual samples except two (positions 3874 and 4138) that each occurred in two samples (Fig. 4B). Since PvIno1 is effectively undetectable in HHC VT samples or any viromes (except one UC virome), robust statistical comparisons of SNP abundance between conditions were not possible (Table 2). Of note, several SNPs occurred within the proposed intergenic region, with that at position 3874 occurring directly within the proposed *ori−* and four bases removed from the strongest hairpin structure. Though sampling was insufficient to make any conclusions here, these SNPs highlight potential evolutionary pressures targeting regulatory loci over coding regions.

## Discussion

By using VT, our work overcomes the limitations of standard ‘omics sequencing to capture low-abundance, host-defined phage populations at high sequence resolution, shifting the study of the IBD phageome from a macro-ecological overview to high-resolution population and evolutionary dynamics. Using *P. vulgatus* as the target host, we identified 43 vOTUs, including BV01-like temperate tailed phages and the first reported filamentous phage, PvIno1, infecting any *Bacteroidaceae* host. Crucially, PvIno1’s near-exclusive detection in CD samples points to it as a promising disease-associated biomarker. Indepth sequence analysis revealed active infection of *P. vulgatus* cells and distinct evolutionary trajectories between phage types and gut conditions.

The bacterial cell surface is the first barrier to phage infection, and is especially challenging for phages of *Bacteroidaceae* species like *P. vulgatus*, which express multiple CPS loci under the control of invertible promoters, generating mixed populations. In BV01-like phages, we identified a direct counter-mechanism to this surface variation: a multi-cassette shufflon that recombinantly generates six isoforms of the carbohydrate-binding tail fiber. CPS profiles dictate phage susceptibility in other *Bacteroidaceae*-phage systems^43–45^, though the BV01-like phage shufflon may allow them to circumvent CPS restriction and maintain a broad host range. While BV01-like phage nucleotide variation is concentrated within structural genes, variation in the PvIno1 genome is largely non-structural, restricted to a handful of SNPs surrounding the predicted *ori−* regulatory region. Although the near-exclusivity of PvIno1 to CD VT samples prevented evolutionary comparison between conditions, these data still suggest a fundamentally different evolutionary trajectory than BV01-like phages, whose tail hypervariability persists across both health and disease.

Beyond enabling phage-host identification and population-level evolutionary analyses, we found that VT provides a window into the active viral lifecycle. Consistent with our prior work observing prophage integration during VT of *Faecalibacterium prausnitzii*^10^, VT again captured new integration events, here involving BV01-like phages. Remarkably, VT also captured active RCR of PvIno1 in *P. vulgatus*, an unanticipated observation given the workflow’s constraints.

Our study was limited by the small sample size of input VLPs, which were pooled within CD and HHC for practicality. Applying VT to individual viromes will be necessary to resolve individual variation or longitudinal dynamics through IBD flares. Profiling a single bacterial host strain further limited our scope. Expanding VT across diverse, related hosts will be required to establish phage host range and broader population structures. At a technical level, VT remains reliant on MDA, which can introduce biases and artifacts^46^. Future bulk VT applications could eliminate MDA by sorting larger cell populations while offsetting sequence resolution loss with deeper sequencing. Finally, while VT circumvents the constraints of viromics to recover low-abundance phages, it also captured a large quantity of sequence that failed annotation to a meaningful degree. This highlights a methodological paradox of VT: by recovering ultra-rare phage diversity, it expands the known virological landscape past the boundaries of biological interpretation.

Ultimately, we have moved beyond overly broad metagenomic phage surveys to resolve the evolutionary trajectories required to understand how distinct phages adapt to the inflamed gut. VT’s power is highlight by its ability to afford characterization of multiple levels of phage biology: host identification, infection dynamics, and patterns of evolution. Understanding fine-scale adaptations will be essential for predicting phageome activities during chronic disease and informing the development of phage therapies to restore gut microbiome homeostasis.

## Methods

### Stool sample & metadata collection

The VLP samples were utilized here as in our previous work^10^. Briefly, stool samples from a cohort of longitudinally sampled Crohn’s disease (CD) patients (*n* = 8) and matched healthy household control (HHC) individuals (*n* = 8) (Table S1), collected at Addenbrooke’s Hospital, University of Cambridge, UK between 2016 and 2019 were used. One to two samples from each individual were used (*n* = 9 CD; *n* = 10 HHC). Of the 9 CD stool samples, six were collected at the time of active inflammation as indicated by fecal calprotectin (≥ 600 μg/mg) and/or serum C-reactive protein (≥ 10 mg/L) levels (**Table S1**). Stool samples were stored at − 80°C.

### Isolation of VLPs & pool preparation

VLPs extractions and pools were prepared as part of our previous work^10^. Approximately 0.2 g of each stool sample was homogenized in a 6X volume of PBS by vortexing for 10 min. Homogenates were incubated at room temperature for 10 min to allow debris to settle. The upper fraction was removed and centrifuged for 10 min at 7,000 x*g* to pellet uncultivated cells. Supernatants were removed and sequentially filtered through 0.45 μm and 0.22 μm filters to obtain the VLP fraction. To make the CD and HHC VLP pools, equal volumes of VLPs were combined within the groups.

### P. vulgatus strain & cultivation

*P. vulgatus* ΔBV01 is a mutant of the type strain (ATCC 8482) generated previously, wherein the BV01 prophage was deleted by homologous recombination, leaving a scarless empty integration site^31^. This strain was used for all experiments.

*P. vulgatus* was cultured anaerobically in a vinyl anaerobic chamber with 70% N_2_, 20% CO_2_, and 10% H_2_ gas mixture. Cultures were grown on Difco Brain Heart Infusion (BHI) agar supplemented with 10% defibrinated sheep blood, or in tryptone-yeast extract-glucose broth (10 g/L tryptone, 5 g/L yeast extract, 2 g/L glucose, 500 mg/L cysteine free base, 100 mM pH 7.2 potassium phosphate, 20 mg/L MgSO4 · 7H2O, 400 mg/L NaHCO3, 80 mg/L NaCl, 0.0008% CaCl2, 0.4 μg/L FeSO4, 1 μg/L menadione, 0.2 mM histidine, 1.9 μM hematin) at 37°C.

### In vitro *enrichment*

Enrichment was performed by adding 500 μL of VLPs from sample HHC-1 to a 5-mL mid-log phase culture of *P. vulgatus* and then incubating overnight. On the subsequent day, the culture was centrifuged at 21,000 x*g* for 2 min and the supernatant passed through a 0.22 μm filter, and 500 μL of filtered supernatant was added to a new mid-log phase culture. This was repeated for a total of three passages. One experimental control was performed in which no VLPs were added on Day 0, but supernatants were still passaged as above. On Day 3, final supernatants were filtered and stored at 4°C for 48 hr prior to DNA preparation for sequencing.

### Viral tagging, flow cytometry & cell sorting

Viral tagging, flow cytometry, and cell sorting of *P. vulgatus* ΔBV01 was performed as previously described^10^, except that tagged cells were sorted in bulk as samples of 1.08 × 10^5^ and 2.04 × 10^5^ cells for CD and HHC experiments, respectively, then split across 12 fractions each (approximately 8,962 and 16,973 cells per fraction), functioning as technical replicates prior to sequencing.

Briefly, SYTO 9-stained *P. vulgatus* cells and SYTO 62-stained VLP pools were combined in equal volumes, incubated on ice for 20 min, and washed to remove unbound VLPs. Flow cytometry and cell sorting were conducted on a BD FACSAria III (70 μm nozzle, 70 psi, ~ 10,000 events/s). Gating for singlet bacterial cells followed established procedures^10^, using unstained and SYTO-9–only controls to define autofluorescence thresholds. Host–phage complexes corresponding to the top 10% most fluorescent SYTO-9^+^/SYTO-62^+^ single cells were bulk-sorted into microfuge tubes containing 100 μL TE buffer. Sorted samples were transported on dry ice, partitioned into 12 equal fractions (~ 8 μL) and stored at − 80°C prior to DNA extraction.

### DNA extraction & amplification

For VLP preparation from enrichment cultures, 5 mL cultures were centrifuged at 2,500 x*g* for 5 min, the supernatant was collected and filtered with a 0.22 um PVDF filter. 800 uL of supernatant was treated to lyse bacterial cells and remove any non-VLP DNA. To the supernatant was added 20 uL Turbo DNase I (Thermo Fisher AM2238), 4 uL Baseline Zero DNase (Lucigen DB0715K), 80 uL chicken egg white ysozyme solution (EMD Millipore 71412-3), and 80 uL RNase A (NEB T3018) with 108 uL Turbo DNase Buffer. This was incubated at 37°C overnight, and 10.8 uL of 0.5M EDTA was added to stop the reaction. The treated VLP preparation was then treated with 80 uL 10% SDS and 40 uL Proteinase K (Qiagen 1018332) overnight at 56°C. DNA was cleaned using a standard phenol-chloroform (VWR V0883) protocol and DNA precipitation.

For each bulk VT sample, DNA isolation and multiple displacement amplification (MDA) were performed using a modified alkaline lysis protocol previously described^10,47^. Briefly, sorted cell samples (~ 8 μL) were lysed in a KOH/EDTA solution at 30°C for 30 min and neutralized with Tris-HCl (pH 4.0). To eliminate background DNA contamination prior to amplification, MDA master mix reagents were UV-irradiated, followed by an enzymatic decontamination step utilizing the intrinsic 3’–5’ exonuclease activity of Phi29 DNA polymerase prior to dNTP addition. Decontaminated master mix containing Phi29 DNA polymerase (NEB) and exo-resistant random hexamers (MCLAB) was added to each neutralized sample. MDA reactions were incubated at 30°C for 12 h and terminated by heat inactivation at 65°C for 10 min. Amplified DNA was diluted with TE buffer and quantified using the Qubit dsDNA HS Assay Kit (Invitrogen).

### Sequencing

Purified and/or amplified DNA for enrichments and VT were tagmented using the Nextera DNA Library Preparation kit (Illumina) as previously described^48^. This was followed by PCR-mediated adapter ligation using KAPA HiFi PCR master mix (Roche). The tagmented and indexed DNA libraries were selected for approximately 200bp size using AMPure XP magnetic beads. Equimolar quantities of libraries were run on the Illumina NextSeq 500 platform using a 2 × 150 protocol at the DNA Sequencing Innovation Lab (Washington University School of Medicine).

### Sequence filtering & assembly

Analysis of VT sequence data was carried out using the customized pipeline PvVTPhageFinder (v1.0.0) (https://github.com/xwu35/PvVTPhageFinder). In brief, read quality was assessed using FastQC^49^ (v0.12.1) and MultiQC^50^ (v1.14). Adapters and low-quality reads were removed using Trimmomatic^51^ (v0.39) with parameters “ILLUMINACLIP:resources/adapters.fna:2:30:10:2:True SLIDINGWINDOW:4:20 MINLEN:36”. Minimap2^52^ (v2.28) was used to map reads to the PhiX and human genomes, respectively. Reads mapped to those genomes were removed using SAMtools^53^ (v1.21). After removing PhiX and human contaminants, the reads were subsequently mapped back to the *P. vulgatus* ΔBV01 genome using Minimap2^52^ (v2.28). Reads that matched the genome with an identity of ≥ 98% were removed using the filter mode from CoverM^54^. The remaining reads were individually assembled across VT samples by condition using MEGAHIT^55^ (v1.2.9) with default parameters. Contigs with a length ≥1kb were used to conduct a BLASTn^33^ (v2.14.0) search against the host genome with an e-value of 1e-3. Average nucleotide identity (ANI) and alignment fraction (AF) values were calculated from BLASTn results using the anicalc.py script from CheckV^56^ (v1.0.1). Contigs that aligned to the host genome with ANI ≥ 95% and AF ≥ 85% were removed. VT sample Pv_CD_2 did not assemble and was not analyzed due to low sequencing depth (906 initial reads).

Analysis of phage enrichment sequences was performed as in VT with the exception that metaSPAdes^57^ (v4.1.0) was used for assembly with default parameters. Analysis of the input virome for enrichments was performed with a customized pipeline, VLPVirFinder (v1.0.0) (https://github.com/xwu35/VLPVirFinder). Similar to the VT analysis, Trimmomatic^51^ (v0.39), Minimap2^52^ (v2.28), and SAMtools^53^ (v1.21) were used to remove adapters, low-quality reads, PhiX, and human reads. Contigs were assembled with metaSPAdes^57^ (v4.1.0) using default parameters. Contigs with a length ≥1kb were analyzed to identify those with viral origin using (1) geNomad (v1.11.1)^34^ end-to-end module (--sensitivity 7.5 --enable-score-calibration); (2) VirSorter2^58^ (v2.2.4) (--keep-original-seq --include-groups dsDNAphage,ssDNA --min-length 1000 --min-score 0.5); (3) Cenote-Taker3 (v3.4.3)^59^ (-p F -db {virion,rdrp,dnarep}). Contigs were considered viral if they were identified by at least one of the methods. CheckV^56^ (v1.0.3) was used to evaluate the quality of the identified viral contigs. No proviruses were identified by geNomad and CheckV in this sample.

Resulting contigs were manually filtered using BLASTn^33^ (v2.17.0+) to remove remaining host contigs resulting from transposon movement or other recombination events within the *P. vulgatus* ΔBV01 genome. These events were identified by complete but noncontiguous coverage of contigs aligning to the host genome and the aligning contigs were removed.

To increase phage genome quality, sample reads were mapped to contig fragments using strobealign (v0.17.0)^60^ and those alignments were utilized for binning through SemiBin2 (v2.2.1)^61^ and vRhyme (v1.1.0)^62^. Despite these efforts, no high-quality bins were identified for either the enrichment or VT datasets.

### Viral annotation

Annotation of viral contigs was performed using pharokka^63^ (v1.9.1) with PyHMMER^64^ (v0.12.0) supplemented with the Pfam database^65^. For vOTU_0, predicted protein sequences were clustered and mapped to the BV01 reference proteome with MMseqs2^66^ (v15.fac81) using the easy-search workflow with a minimum sequence identity threshold of 30% and 80% query coverage. For select proteins of interest, domains were predicted with the InterPro Scan (v108.0) webtool^67^ and structural motifs with AlphaFold2 via ColabFold^68^ (v1.6.1).

Viral taxonomy was assigned by geNomad^34^ (v1.11.2) in end-to-end “lenient” mode. To identify relationships with known phages, vConTACT2^32^ (v0.11.3) was employed using DIAMOND^69^ (v2.1.24) and the ProkaryoticViralRefSeq211-Merged database^70^. Sequences were further compared to the RefSeq Viral 233 database using BLASTn^33^ (v2.17.0+) and matches were called if percent length aligned (PLA) ≥ 20%. PLA was calculated by merging overlapping BLASTn^33^ (v2.17.0+) alignments for query-subject pairs to find the total unique aligned length, then dividing that sum by the query sequence’s full length.

Hairpin loops were predicted by scanning sequences with a 100-bp sliding window and a 1-bp step size, allowing for one mismatch within defined stem (6–15 bp) and loop (4–12 bp) length constraints. Putative structures were subsequently evaluated via thermodynamic stability, where the estimated Gibbs free energy was calculated using an empirical heuristic combining structural penalties with base pairing stability. Helix initiation was assigned a fixed penalty of + 1.96 kcal/mol, while an unfavorable loop entropy penalty scaled logarithmically with loop length (2.44 × length). Stabilizing energy contributions were then subtracted for each exact match within the stem, weighting G-C pairs at −2.5 kcal/mol and A-T pairs at −1.0 kcal/mol. Finally, overlapping predictions were removed to retain only the most thermodynamically stable conformation for each genomic locus.

All protein-based contig alignments were generated using clinker^71^ (v0.0.29).

To search for CRISPR protospacers against filamentous phages in vOTU_10, genomes were queried against the JGI Global CRISPR Spacer Database by BLASTn (v2.17.0+)^33^ using blastn-short, although no matches were found.

### vOTU clustering

Viral contigs were clustered into vOTUs based on a 95% average nucleotide identity (ANI) threshold with a reference and query coverage minimum of 85%^72^, using the align and cluster functions within vClust^73^ (v2.3.1) using the CD-HIT algorithm and a threshold of 0.95 ANI. Networks were built using ANI values and visualized in Cytoscape^74^ (v3.10.2).

### Differential abundance analysis

Viral abundance was quantified by mapping filtered reads to vOTU-representative contigs (the largest contig within the vOTU, unless indicated otherwise) using strobealign^60^ (v0.17.0) in abundance estimation mode and SAMtools idxstats^53^ (v1.19). Raw counts were converted to transcripts per million (TPM) to adjust for contig length and sequencing depth. A noise threshold of 0.5 TPM was applied. After filtering rare vOTUs (< 5% prevalence), differential abundance across cohorts was assessed via two-sided Mann-Whitney U tests with Benjamini-Hochberg FDR correction (q < 0.05).

### Phylogenetic analysis

To construct the vOTU_0 phylogenetic tree, 18 phage contigs > 48 kb from the assembled VT fractions were compared against 17 reference genomes from the *Salyersviridae* family. Six core functional viral proteins were selected as phylogenetic markers based on their conserved presence across the group: portal protein, major head protein, head maturation protease, single-strand DNA binding protein, RNA polymerase sigma factor, and RecE-like recombination exonuclease. Protein sequences were extracted from pharokka outputs and each marker set was aligned using MAFFT^75^ (v7.526) with the --auto parameter. The six resulting alignments were concatenated side-by-side. The phylogenetic tree was inferred using FastTree^76^ (v2.2.11) (Price et al., 2010) using the Le-Gascuel evolutionary model with a Gamma distribution to account for varying rates of evolution across sites. The tree was rooted on phage BF01 from *Bacteroides finegoldii* CL09T03C10^31^ and visualized in FigTree (v1.4.4) (http://tree.bio.ed.ac.uk/software/figtree). Branch support was assessed using Shimodaira-Hasegawa-like local support values.

### Quantification of phage-bacteria dynamics

To evaluate phage-bacterial host dynamics, human-filtered reads from VT fractions and enrichments were aligned to hybrid references containing the *P. vulgatus* genome and target phage genome sequences. Alignments were performed using BWA-MEM^77^ (v0.7.18) with the -Y flag enabled to preserve soft-clipping of supplementary alignments. High-confidence, uniquely mapped reads were retained by filtering out alignments with a mapping quality score of zero.

The per-base nucleotide coverage was extracted using SAMtools depth^53^ (v1.21) and the average coverage depth of phage contigs was calculated. The relative copy number ratio was defined as the average depth of the phage divided by the average depth of the bacterial chromosome.

Prophage integration events were identified through discordant read pairs or chimeric split reads possessing a Supplementary Alignment (SA) tag crossing a phage-host boundary. Total integration evidence was calculated as the sum of these unique discordant pairs and split reads. Lysogeny rates were calculated by normalizing the total integration evidence to the local coverage depth of reads or pairs spanning the integration locus within a 500 bp window.

Phage genome circularization was quantified using a 2kb (for vOTU_0 phages) or 500bp (for vOTU_10 phages) windows at the 5’ and 3’ ends of the vOTU reference genome. Circularization-specific evidence was defined as split reads and paired-end reads directly bridging the 5′ and 3′ windows. The circularization rate was calculated as the ratio of circular evidence to total terminal coverage.

### Single nucleotide polymorphism analysis & genome wide association

Human-filtered reads were mapped against target reference consensus contigs using BWA-MEM^77^ (v0.7.18). Subclonal variants and frequencies were identified using LoFreq^78^ (v2.1.5) with a minimum quality score of 20, minimum coverage of 100, and a minimum minor allele frequency of 1%, and were subsequently annotated by SnpEff^79^ (v5.4c). To calculate population differentiation and diversity metrics along phage genomes, multi-cohort alignment data were combined using SAMtools mpileup^53^ with disabled base alignment quality calculations and a minimum base quality score of 20. Nucleotide diversity and selection profiles (*P_N_/P_S_*) were quantified from the pileup data across matching 200-bp sliding windows and 50-bp steps. *P_N_/P_S_* was calculated using Haldane’s continuity correction. A pseudocount of 0.5 was added to both nonsynonymous and synonymous mutation counts before normalizing each count by its respective target site length to determine the final adjusted ratio.

Individual nucleotide variants and allele frequencies (AF) were called for reference-mapped and/or self-mapped contigs. For self-mapped analyses, contig coordinates were mapped back to the reference genome using the nucmer tool within MUMmer^80^ (v3.1) with default parameters. Within-sample nucleotide diversity was calculated per position using Nei’s unbiased estimator, which measures the probability that two randomly selected reads at a site carry different alleles, corrected for local read depth. Per-site diversity values were then averaged across all evaluated genomic positions to yield overall nucleotide diversity. Variant density was defined as total mutations per base pair, and *P_n_/P_s_* selective constraints were established using a pseudocount-corrected ratio (0.05) of missense to synonymous mutations.

### Recombination analysis

To profile the region of recombination in vOTU_0 phage contigs, contigs were aligned to the BV01 reference region spanning the upstream recombinase, region of recombination, and the downstream tail fiber gene using BLASTn^33^ (v2.17.0+). The endpoints of all overlapping fragments were used to define consensus boundaries and identify a shared 10-bp core motif separating all cassettes. To quantify cassette-gene junctions in read data, we aligned reads to the BV01 reference, filtered for those covering the 10-bp of the tail fiber gene directly adjacent to the region of recombination, and read the next 10 bases on the read to identify which forward or inverted cassette was linked to the annotated tail gene.

### Rarefaction curves

Richness rarefaction curves were generated using trimmed reads from VT and enrichments, and the longest contig from each vOTU cluster. The trimmed reads were subsampled using the reformt.sh script from BBMap (v39.01; https://escholarship.org/uc/item/1h3515gn) and mapped to the vOTU representative contigs to calculate their abundance using CoverM^54^ (v0.7.0) with the ‘-m count --min-read-percent-identity 0.95 --min-read-aligned-percent 0.75 --min-covered-fraction 0’ parameters. The observed index was calculated using the R package vegan (v2.7-2) (https://cran.r-project.org/web/packages/vegan/index.html).

VT coverage rarefaction curves were generated by mapping the subsampled trimmed reads to all VT viral contigs using Bowtie2^81^ (v2.4.2) with the “--very-sensitive” flag. The number of covered bases were obtained using SAMtools^53^ (v1.21). The percentage of covered bases were calculated by dividing the number of covered bases by the total length of the viral contigs.

### Use of AI tools

Google Gemini was used during manuscript preparation to refine text for concision and clarity, and to assist in refining Python scripts for figure plotting for downstream assembly in Inkscape. All AI-assisted text, code, and visual outputs were critically reviewed, verified, and approved by the authors, who take full responsibility for the manuscript’s contents.

## Supplementary Material

This is a list of supplementary files associated with this preprint. Click to download.

- suppfiguresv8.docx
- supptablesv2.xlsx

## Figures and Tables

**Table 1.** Prevalence and abundance of vOTU_0 and vOTU_0-similar contigs.

Group	Source	Contig count	Total length	Metric	Cohort viromes (*n* = 19)	VT (*n* = 23)
vOTU_0	VT	73	1,455.9 kb	Prevalence	57.9%	100.0%
				RPKM	137.2 ± 231.4	691.6 ± 0.04

vOTU_0-similar	Metaviromes	5	67.7 kb	Prevalence	89.5%	78.3%
				RPKM	11,837.6 ± 4,940.5	0.78 ± 0.87

**Table 2.** Prevalence and abundance of vOTU_10 contigs by condition.

Dataset	Condition	Samples	Prevalence	RPKM	Prevalence *p*-value^†^	Abundance *p*-value^††^
VT	CD	11	100.0%	583.9 ± 69.8	3.3×10^−4^	3.2×10^−5^
	HHC	12	25.0%	0.01 ± 0.02		

Cohort viromes	CD	9	0.0%	0.0 ± 0.0	1.0	1.0
	HHC	10	0.0%	0.0 ± 0.0		

Large cohort	CD	37	0.0%	0.0 ± 0.0	1.0 (All pairwise comparisons)	≥0.32 (All pairwise comparisons)
viromes	HHC	66	0.0%	0.0 ± 0.0		
	UC	66	1.5%	56.8 ± 461.4		

† Fisher’s exact test;

†† Mann Whitney U Test

## Data Availability

All shotgun sequencing reads have been deposited to the European Nucleotide Archive (ENA) at EMBL-EBI under accession number PRJEB123053 and will be made public prior to publication. Additional datasets are available from the corresponding author upon reasonable request during the review process.

## Code availability

The primary pipeline for viral tagging analysis (PvVTPhageFinder) is accessible at https://github.com/xwu35/PvVTPhageFinder. Additional Python scripts are available from the corresponding author upon reasonable request.

## References

[1] Nishiyama H, Endo H, Blanc-Mathieu R, Ogata H (2020) Ecological Structuring of Temperate Bacteriophages in the Inflammatory Bowel Disease-Affected Gut. Microorganisms 8:1663 10.3390/microorganisms8111663 PMC7692956 33121006

[2] Clooney AG (2019) Whole-Virome Analysis Sheds Light on Viral Dark Matter in Inflammatory Bowel Disease. Cell Host Microbe 26:764–778e5 10.1016/j.chom.2019.10.009 31757768

[3] Norman JM (2015) Disease-specific alterations in the enteric virome in inflammatory bowel disease. Cell 160:447–460 10.1016/j.cell.2015.01.002 PMC4312520 25619688

[4] Shan Y, Lee M, Chang EB (2022) The Gut Microbiome and Inflammatory Bowel Diseases. Annu Rev Med 73:455–468 10.1146/annurev-med-042320-021020 PMC10012812 34555295

[5] Xiao Y (2025) The role of bacteriophage in inflammatory bowel disease and its therapeutic potential. Crit Rev Microbiol 51:1231–1245 10.1080/1040841X.2025.2492154 40219702

[6] Pye HV, Krishnamurthi R, Cook R, Adriaenssens EM (2024) Phage diversity in One Health. Essays Biochem 68:607–619 10.1042/EBC20240012 PMC12055037 39475220

[7] Nie W (2024) Advances in phage–host interaction prediction: in silico method enhances the development of phage therapies. Brief Bioinform 25:bbae117 10.1093/bib/bbae117 PMC10981677 38555471

[8] Shang J (2025) From genomic signals to prediction tools: a critical feature analysis and rigorous benchmark for phage–host prediction. Brief Bioinform 26:bbaf626 10.1093/bib/bbaf626 PMC12641615 41283812

[9] Malajczuk CJ (2026) Towards accurate artificial intelligence models for strain-level phage–host prediction. Brief Bioinform 27:bbag085 10.1093/bib/bbag085 PMC12936788 41744225

[10] Campbell DE (2025) Single cell viral tagging of Faecalibacterium prausnitzii reveals rare bacteriophages omitted by other techniques. Gut Microbes 17:2526719 10.1080/19490976.2025.2526719 PMC12323421 40754853

[11] Deng L (2014) Viral tagging reveals discrete populations in Synechococcus viral genome sequence space. Nature 513:242–245 10.1038/nature13459 25043051

[12] Džunková M (2019) Defining the human gut host–phage network through single-cell viral tagging. Nat Microbiol 4:2192–2203 10.1038/s41564-019-0526-2 31384000

[13] Deng L (2012) Contrasting Life Strategies of Viruses that Infect Photo- and Heterotrophic Bacteria, as Revealed by Viral Tagging. mBio 3. 10.1128/mbio.00373-12 PMC3487772 23111870

[14] Hyman P (2025) Are You My Host? An Overview of Methods Used to Link Bacteriophages with Hosts. Viruses 17:65 10.3390/v17010065 PMC11769497 39861854

[15] Schirmer M (2018) Dynamics of metatranscription in the inflammatory bowel disease gut microbiome. Nat Microbiol 3:337–346 10.1038/s41564-017-0089-z PMC6131705 29311644

[16] Liu L (2022) Bacteroides vulgatus attenuates experimental mice colitis through modulating gut microbiota and immune responses. Front Immunol 13:1036196 10.3389/fimmu.2022.1036196 PMC9750758 36531989

[17] Li S (2021) Evaluation of the Effects of Different Bacteroides vulgatus Strains against DSS-Induced Colitis. J. Immunol. Res. 9117805 (2021) 10.1155/2021/9117805 PMC8181088 34195297

[18] Ó, Cuív P (2017) The gut bacterium and pathobiont *Bacteroides vulgatus* activates NF-κB in a human gut epithelial cell line in a strain and growth phase dependent manner. Anaerobe 47:209–217 10.1016/j.anaerobe.2017.06.002 28583864

[19] Bamba T, Matsuda H, Endo M, Fujiyama Y (1995) The pathogenic role of Bacteroides vulgatus in patients with ulcerative colitis. J Gastroenterol 30:45–47 8563888

[20] Lucke K, Miehlke S, Jacobs E, Schuppler M (2006) Prevalence of Bacteroides and Prevotella spp. in ulcerative colitis. J Med Microbiol 55:617–624 10.1099/jmm.0.46198-0 16585651

[21] Mills RH (2022) Multi-omics analyses of the ulcerative colitis gut microbiome link Bacteroides vulgatus proteases with disease severity. Nat Microbiol 7:262–276 10.1038/s41564-021-01050-3 PMC8852248 35087228

[22] Breeling JL, Onderdonk AB, Cisneros RL, Kasper DL (1988) Bacteroides vulgatus outer membrane antigens associated with carrageenan-induced colitis in guinea pigs. Infect Immun 56:1754–1759 10.1128/iai.56.7.1754-1759.1988 PMC259473 3384476

[23] Qi X (2019) Gut microbiota-bile acid-interleukin-22 axis orchestrates polycystic ovary syndrome. Nat Med 25:1225–1233 10.1038/s41591-019-0509-0 PMC7376369 31332392

[24] Yoshida N (2018) Bacteroides vulgatus and Bacteroides dorei Reduce Gut Microbial Lipopolysaccharide Production and Inhibit Atherosclerosis. Circulation 138:2486–2498 10.1161/CIRCULATIONAHA.118.033714 30571343

[25] Liu Z (2026) IDDF2026-ABS-0034 Gut microbiota-derived bacteroides vulgatus regulates diabetic atherosclerotic plaque stability via the 4. 10.1136/gutjnl-2026-IDDF.53. - methylcatechol-notch pathway

[26] Qi Z (2025) The gut microbiota-bile acid-TGR5 axis orchestrates platelet activation and atherothrombosis. Nat Cardiovasc Res 4:584–601 10.1038/s44161-025-00637-x 40217125

[27] Maerz JK (2017) Outer membrane vesicles blebbing contributes to B. vulgatus mpk-mediated immune response silencing. Gut Microbes 9:1–12 10.1080/19490976.2017.1344810 PMC5914909 28686482

[28] Molinari R, Merendino N, Costantini L (2022) Polyphenols as modulators of pre-established gut microbiota dysbiosis: State-of-the-art. BioFactors 48:255–273 10.1002/biof.1772 PMC9291298 34397132

[29] Vendrell-Fernández S (2025) Incomplete lytic cycle of a widespread Bacteroides bacteriophage leads to the formation of defective viral particles. PLoS Biol 23:e3002787 10.1371/journal.pbio.3002787 PMC12135933 40163458

[30] Benler S (2018) A diversity-generating retroelement encoded by a globally ubiquitous *Bacteroides* phage. Microbiome 6:191 10.1186/s40168-018-0573-6 PMC6199706 30352623

[31] Campbell DE (2020) Infection with Bacteroides phage BV01 alters the host transcriptome and bile acid metabolism in a common human gut microbe. Cell Rep 32 10.1016/j.celrep.2020.108142 PMC8354205 32937127

[32] Jang B (2019) Taxonomic assignment of uncultivated prokaryotic virus genomes is enabled by gene-sharing networks. Nat Biotechnol 37:632–639 10.1038/s41587-019-0100-8 31061483

[33] Camacho C (2009) BLAST+: architecture and applications. BMC Bioinformatics 10:421 10.1186/1471-2105-10-421 PMC2803857 20003500

[34] Camargo AP (2024) Identification of mobile genetic elements with geNomad. Nat Biotechnol 42:1303–1312 10.1038/s41587-023-01953-y PMC11324519 37735266

[35] Zhang S, Chu M, Sun X (2025) The arms race in bacteria-phage interaction: deciphering bacteria defense and phage anti-defense mechanisms through metagenomics. Front Microbiol 16 10.3389/fmicb.2025.1687307 PMC12568517 41170433

[36] Binga EK, Lasken RS, Neufeld JD (2008) Something from (almost) nothing: the impact of multiple displacement amplification on microbial ecology. ISME J 2:233–241 10.1038/ismej.2008.10 18256705

[37] Kim K-H, Bae J-W (2011) Amplification Methods Bias Metagenomic Libraries of Uncultured Single-Stranded and Double-Stranded DNA Viruses. Appl Environ Microbiol 77:7663–7668 10.1128/AEM.00289-11 PMC3209148 21926223

[38] Roux S (2016) Towards quantitative viromics for both double-stranded and single-stranded DNA viruses. PeerJ 4:e2777 10.7717/peerj.2777 PMC5168678 28003936

[39] Parras-Moltó M, Rodríguez-Galet A, Suárez-Rodríguez P, López-Bueno A (2018) Evaluation of bias induced by viral enrichment and random amplification protocols in metagenomic surveys of saliva DNA viruses. Microbiome 6:119 10.1186/s40168-018-0507-3 PMC6022446 29954453

[40] Nelms BL, Labosky PA (2011) A predicted hairpin cluster correlates with barriers to PCR, sequencing and possibly BAC recombineering. Sci Rep 1:106 10.1038/srep00106 PMC3255507 22355623

[41] Ross MG (2013) Characterizing and measuring bias in sequence data. Genome Biol 14:R51 10.1186/gb-2013-14-5-r51 PMC4053816 23718773

[42] Aird D (2011) Analyzing and minimizing PCR amplification bias in Illumina sequencing libraries. Genome Biol 12:R18 10.1186/gb-2011-12-2-r18 PMC3188800 21338519

[43] Porter NT (2020) Phase-variable capsular polysaccharides and lipoproteins modify bacteriophage susceptibility in Bacteroides thetaiotaomicron. Nat Microbiol 1–12. 10.1038/s41564-020-0746-5 PMC7482406 32601452

[44] Carasso S (2024) Inflammation and bacteriophages affect DNA inversion states and functionality of the gut microbiota. Cell Host Microbe 32:322–334e9 10.1016/j.chom.2024.02.003 PMC10939037 38423015

[45] Shkoporov AN (2021) Long-term persistence of crAss-like phage crAss001 is associated with phase variation in Bacteroides intestinalis. BMC Biol 19:163 10.1186/s12915-021-01084-3 PMC8375218 34407825

[46] Ospino MC (2024) Evaluation of multiple displacement amplification for metagenomic analysis of low biomass samples. ISME Commun 4:ycae024 10.1093/ismeco/ycae024 PMC10945365 38500705

[47] Lawrence D (2022) Single-cell genomics for resolution of conserved bacterial genes and mobile genetic elements of the human intestinal microbiota using flow cytometry. Gut Microbes 14:2029673 10.1080/19490976.2022.2029673 PMC8824198 35130125

[48] Baym M (2015) Inexpensive multiplexed library preparation for megabase-sized genomes. PLoS ONE 10:e0128036 10.1371/journal.pone.0128036 PMC4441430 26000737

[49] Andrews S (2010) FastQC A Quality Control Tool for High Throughput Sequence Data. - References - Scientific Research Publishing. https://www.scirp.org/reference/referencespapers?referenceid=4024153

[50] Ewels P, Magnusson M, Lundin S, Käller M (2016) MultiQC: summarize analysis results for multiple tools and samples in a single report. Bioinformatics 32:3047–3048 10.1093/bioinformatics/btw354 PMC5039924 27312411

[51] Bolger AM, Lohse M, Usadel B (2014) Trimmomatic: a flexible trimmer for Illumina sequence data. Bioinformatics 30:2114–2120 10.1093/bioinformatics/btu170 PMC4103590 24695404

[52] Li H (2021) New strategies to improve minimap2 alignment accuracy. Bioinformatics 37:4572–4574 10.1093/bioinformatics/btab705 PMC8652018 34623391

[53] Danecek P (2021) Twelve years of SAMtools and BCFtools. GigaScience 10, giab008 10.1093/gigascience/giab008 PMC7931819 33590861

[54] Aroney STN (2025) CoverM: read alignment statistics for metagenomics. Bioinformatics 41:btaf147 10.1093/bioinformatics/btaf147 PMC11993303 40193404

[55] Li D, Liu C-M, Luo R, Sadakane K, Lam T-W (2015) MEGAHIT: an ultra-fast single-node solution for large and complex metagenomics assembly via succinct de Bruijn graph. Bioinformatics 31:1674–1676 10.1093/bioinformatics/btv033 25609793

[56] Nayfach S (2021) CheckV assesses the quality and completeness of metagenome-assembled viral genomes. Nat Biotechnol 39:578–585 10.1038/s41587-020-00774-7 PMC8116208 33349699

[57] Nurk S, Meleshko D, Korobeynikov A, Pevzner PA (2017) metaSPAdes: a new versatile metagenomic assembler. Genome Res 27:824–834 10.1101/gr.213959.116 PMC5411777 28298430

[58] Guo J (2021) VirSorter2: a multi-classifier, expert-guided approach to detect diverse DNA and RNA viruses. Microbiome 9:37 10.1186/s40168-020-00990-y PMC7852108 33522966

[59] Tisza MJ, Belford AK, Domínguez-Huerta G, Bolduc B, Buck CB (2021) Cenote-Taker 2 democratizes virus discovery and sequence annotation. Virus Evol 7:veaa100 10.1093/ve/veaa100 PMC7816666 33505708

[60] Sahlin K (2022) Strobealign: flexible seed size enables ultra-fast and accurate read alignment. Genome Biol 23:260 10.1186/s13059-022-02831-7 PMC9753264 36522758

[61] Pan S, Zhao X-M, Coelho LP (2023) SemiBin2: self-supervised contrastive learning leads to better MAGs for short- and long-read sequencing. Bioinformatics 39:i21–i29 10.1093/bioinformatics/btad209 PMC10311329 37387171

[62] Kieft K, Adams A, Salamzade R, Kalan L, Anantharaman K (2022) vRhyme enables binning of viral genomes from metagenomes. Nucleic Acids Res 50:e83 10.1093/nar/gkac341 PMC9371927 35544285

[63] Bouras G (2023) Pharokka: a fast scalable bacteriophage annotation tool. Bioinformatics 39:btac776 10.1093/bioinformatics/btac776 PMC9805569 36453861

[64] Larralde M, Zeller G (2023) PyHMMER: a Python library binding to HMMER for efficient sequence analysis. Bioinformatics 39:btad214 10.1093/bioinformatics/btad214 PMC10159651 37074928

[65] Mistry J (2021) Pfam: The protein families database in 2021. Nucleic Acids Res 49:D412–D419 10.1093/nar/gkaa913 PMC7779014 33125078

[66] Steinegger M, Söding J (2017) MMseqs2 enables sensitive protein sequence searching for the analysis of massive data sets. Nat Biotechnol 35:1026–1028 10.1038/nbt.3988 29035372

[67] Blum M (2025) InterPro: the protein sequence classification resource in 2025. Nucleic Acids Res 53:D444–D456 10.1093/nar/gkae1082 PMC11701551 39565202

[68] Mirdita M (2022) ColabFold: making protein folding accessible to all. Nat Methods 19:679–682 10.1038/s41592-022-01488-1 PMC9184281 35637307

[69] Buchfink B, Xie C, Huson DH (2015) Fast and sensitive protein alignment using DIAMOND. Nat Methods 12:59–60 10.1038/nmeth.3176 25402007

[70] Brister JR, Ako-adjei D, Bao Y, Blinkova O (2015) NCBI Viral Genomes Resource. Nucleic Acids Res 43:D571–D577 10.1093/nar/gku1207 PMC4383986 25428358

[71] Gilchrist CLM, Chooi Y-H (2021) clinker & clustermap.js: automatic generation of gene cluster comparison figures. Bioinformatics 37:2473–2475 10.1093/bioinformatics/btab007 33459763

[72] Roux S (2019) Minimum Information about an Uncultivated Virus Genome (MIUViG). Nat Biotechnol 37:29–37 10.1038/nbt.4306 PMC6871006 30556814

[73] Zielezinski A (2025) Ultrafast and accurate sequence alignment and clustering of viral genomes. Nat Methods 22:1191–1194 10.1038/s41592-025-02701-7 PMC12168504 40374946

[74] Shannon P (2003) Cytoscape: a software environment for integrated models of biomolecular interaction networks. Genome Res 13:2498–2504 10.1101/gr.1239303 PMC403769 14597658

[75] Katoh K, Standley DM (2013) MAFFT Multiple Sequence Alignment Software Version 7: Improvements in Performance and Usability. Mol Biol Evol 30:772–780 10.1093/molbev/mst010 PMC3603318 23329690

[76] Price MN, Dehal PS, Arkin AP (2010) FastTree 2 – pproximately maximum-likelihood trees for large alignments. PLoS ONE 5:e9490 10.1371/journal.pone.0009490 PMC2835736 20224823

[77] Li H, Durbin R (2009) Fast and accurate short read alignment with Burrows-Wheeler transform. Bioinformatics 25:1754–1760 10.1093/bioinformatics/btp324 PMC2705234 19451168

[78] Wilm A (2012) LoFreq: a sequence-quality aware, ultra-sensitive variant caller for uncovering cell-population heterogeneity from high-throughput sequencing datasets. Nucleic Acids Res 40:11189–11201 10.1093/nar/gks918 PMC3526318 23066108

[79] Cingolani P (2012) A program for annotating and predicting the effects of single nucleotide polymorphisms, SnpEff. Fly (Austin) 6:80–92 10.4161/fly.19695 PMC3679285 22728672

[80] Kurtz S (2004) Versatile and open software for comparing large genomes. Genome Biol 5:R12 10.1186/gb-2004-5-2-r12 PMC395750 14759262

[81] Langmead B, Salzberg SL (2012) Fast gapped-read alignment with Bowtie 2. Nat Methods 9:357–359 10.1038/nmeth.1923 PMC3322381 22388286

